# Glioblastoma stem cells induce quiescence in surrounding neural stem cells via Notch signaling

**DOI:** 10.1101/gad.336917.120

**Published:** 2020-12-01

**Authors:** Katerina Lawlor, Maria Angeles Marques-Torrejon, Gopuraja Dharmalingham, Yasmine El-Azhar, Michael D. Schneider, Steven M. Pollard, Tristan A. Rodríguez

**Affiliations:** 1 National Heart and Lung Institute, Imperial College London, London W12 0NN, United Kingdom;; 2 Centre for Regenerative Medicine, Edinburgh Cancer Research UK Centre, University of Edinburgh, Edinburgh EH16 4UU, United Kingdom;; 3 MRC London Institute of Medical Sciences, Institute of Clinical Sciences, Imperial College London, London W12 0NN, United Kingdom

**Keywords:** neural stem cells, quiescence, glioblastoma, Notch, cell competition

## Abstract

There is increasing evidence demonstrating that adult neural stem cells (NSCs) are a cell of origin of glioblastoma. Here we analyzed the interaction between transformed and wild-type NSCs isolated from the adult mouse subventricular zone niche. We found that transformed NSCs are refractory to quiescence-inducing signals. Unexpectedly, we also demonstrated that these cells induce quiescence in surrounding wild-type NSCs in a cell–cell contact and Notch signaling-dependent manner. Our findings therefore suggest that oncogenic mutations are propagated in the stem cell niche not just through cell-intrinsic advantages, but also by outcompeting neighboring stem cells through repression of their proliferation.

## Abstract (precis)

In this study from Lawlor et al., the authors sought to understand the cell of origin for glioblastoma, and analyzed the interaction between transformed and wild-type neural stem cells (NSCs) isolated from the adult mouse subventricular zone niche. Their findings suggest that oncogenic mutations are propagated in the stem cell niche not just through cell-intrinsic advantages, but also by outcompeting neighboring stem cells through repression of their proliferation.

In the adult mammalian brain, neural stem cells (NSCs) can be primarily found in specific neurogenic regions. In rodents these include the subgranular zone (SGZ) of the dentate gyrus (DG) and the subventricular zone (SVZ), which lines the lateral ventricles (Doetsch et al. 1999; Sanai et al. 2004; Quiñones-Hinojosa et al. 2006). In the SVZ niche NSCs coexist in both quiescent and activated states (Quiñones-Hinojosa et al. 2006). Tight regulation of the switch between these two states is vital to ensure that the pool of NSCs does not accumulate DNA damage or become exhausted with time, and therefore understanding how this is achieved is of great interest. The feedback mechanisms that exist to regulate the balance of stem cell activation and quiescence during normal homeostasis remain poorly understood.

Adult NSCs are believed to be a cell of origin in certain brain tumors. One possibility is that these cells accumulate driver mutations over time that compromise the normal controls on their proliferation and migration. These transformed NSCs then escape the niche and acquire further mutations resulting in tumor formation. This has been hypothesized to be the case in glioblastoma (GBM), the most aggressive form of malignant glioma (Louis et al. 2007; Rispoli et al. 2014). In particular, the discovery of a subpopulation of cells within GBM tumors with stem cell characteristics, known as glioblastoma stem cells (GSCs), has lent weight to this notion. This GSC population shares many common features with adult NSCs, including expression of stem and progenitor cell markers, self-renewal capacity, and the ability to generate multilineage progeny (Singh et al. 2004; Cheng et al. 2013). Importantly, GSCs are also able to generate tumors in mice that recapitulate all the classical features of GBM, even when injected in low numbers (Galli et al. 2004; Singh et al. 2004).

Adult NSCs can be isolated from the SVZ of murine brains and cultured as adherent cultures under conditions that promote symmetric self-renewal and prevent differentiation (Conti et al. 2005; Pollard et al. 2006). Furthermore, by using NSCs engineered with oncogenic drivers, these in vitro culture systems can be useful as a model of brain tumor development to understand cellular transformation. Here we exploit this system to understand how transformed NSCs influence wild-type NSCs.

## Results and Discussion

### Transformed NSCs are refractory to quiescence-inducing signals

**Figure 1.**
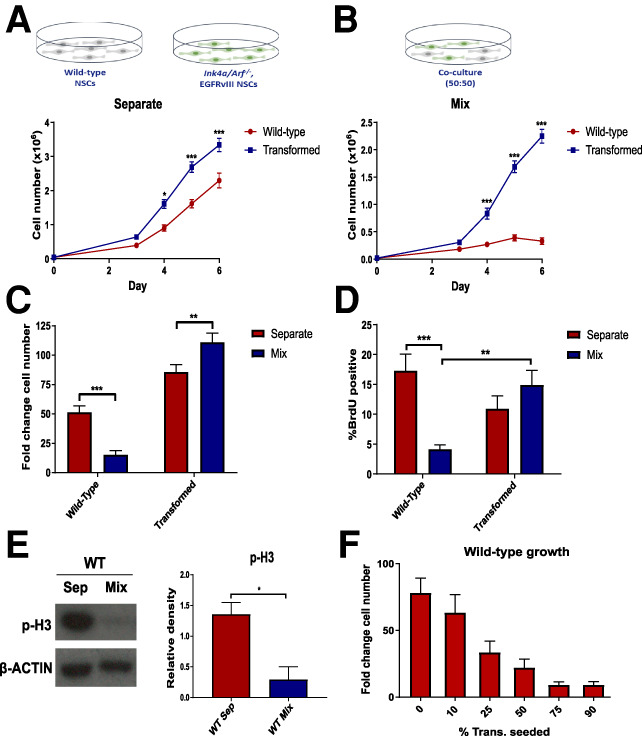
WT-NSCs show reduced proliferation in the presence of transformed NSCs. (*A*,*B*) Growth curves of WT and IE-NSCs cultured separately (*A*) or cocultured (*B*) over 6 d. *N* > 10, ANOVA followed by Sidak's multiple comparisons test. (*C*) Quantification of fold change in cell number relative to seeding density for each culture condition. *N* > 10, ANOVA followed by Sidak's multiple comparisons test. (*D*) Proportion of BrdU-positive cells in each culture condition at day 5 following a 2-h BrdU chase. *N* = 10, ANOVA followed by Sidak's multiple comparisons test. (*E*) Representative immunoblot for phospho-histone-3 (p-H3) in WT-NSCs sorted at day 5 and quantification of p-H3 expression relative to β-actin. *N* = 3, Student's paired *t*-test. (*F*) Fold change cell number of WT-NSCs cultured in cocultures with varying proportions of IE-NSCs. *N* = 3.

To understand how transforming mutations affect the quiescent or activation status of NSCs, we compared NSCs derived from wild-type mice (WT-NSCs) with those isolated from *Ink4a/Arf^−/−^* mice and transduced with a retrovirus expressing the EGFRvIII mutation (IE-NSCs) (Bruggeman et al. 2007). This combination of mutations is frequently observed in human GBM (Crespo et al. 2015) and induces transformation in NSCs, as indicated by their ability to generate tumors in vivo that recapitulate many of the features of human GBM tumors (Bruggeman et al. 2007; Marques-Torrejon et al. 2018). We first compared the proliferative potential of both these cell types. We observed that, when grown in conditions that promote the proliferative NSC state (Pollard et al. 2006), IE-NSCs displayed a small growth advantage (Fig. 1A; Supplemental Fig. S1A,B). We next investigated whether IE-NSCs have a similar response to quiescence-inducing signals when compared with WT-NSCs. BMP signaling is known to induce a reversible quiescent-like state in hippocampal and embryonic stem cell-derived NSCs (Mira et al. 2010; Martynoga et al. 2013). For this reason, we treated WT and IE-NSCs with 50 ng/mL BMP4. We observed that although this induced similar levels of SMAD1/5/8 phosphorylation in both cell types (Supplemental Fig. S1C) and efficiently suppressed the proliferation of WT-NSCs, it had little effect on the growth of the transformed cells (Supplemental Fig. S1D). This suggests that IE-NSCs cells are refractory to the induction of quiescence by BMP signaling activation.

### Transformed NSCs suppress the proliferation of surrounding NSCs

The experiments described above allowed us to test the proliferative potential of wild-type and transformed cells as isolated cell populations. However, we postulated that in the niche these cell types are likely to coexist. For this reason, we next studied the proliferation patterns of both cell types in a 50:50 coculture and compared these proliferative patterns with the growth of cell populations in homotypic (separate) cultures.

IE-NSCs were GFP-labeled, allowing us to accurately quantify the relative cell proliferation rates in coculture using flow cytometry. Given transformed cells produce autocrine and paracrine growth factors, these might support increased WT-NSC expansion. Unexpectedly however, we observed that in contrast to the exponential growth observed for both cell types in separate culture, in coculture WT-NSCs showed a significant reduction in the rate of proliferation (Fig. 1B–E; Supplemental Fig. S1E,F). These results suggest that IE-NSCs have an acquired inhibitory effect on the proliferation of WT-NSCs.

To test the above possibility further, cocultures were established with varying proportions of each cell type, ranging from 10% to 90%, and the total number of cells present in coculture was kept constant. Analysis of the fold change in WT-NSC number, which normalizes for variations in WT-NSC numbers at initial plating, revealed that the reduction in their proliferation was directly proportional to the percentage of transformed NSCs present (Fig. 1F), to the extent that, when 75% of the cells in coculture were IE-NSCs, the 25% of WT-NSCs barely increased in number during the 6-d experiment. Interestingly, cleaved Caspase3 expression was also elevated in cocultured WT-NSCs (Supplemental Fig. S1G). However, this increase was only significant from day 5 of coculture when compared with separate cultures. Given that the differences in cell number and BrdU incorporation were apparent from day 3, this suggests that apoptosis may be due to the compromised ability of arrested NSCs to survive long-term in pro-proliferative conditions. Together, these results indicate that IE-NSCs suppress the proliferation of activated WT-NSCs.

### Transformed NSCs induce a quiescent-like state in neighboring wild-type NSCs

**Figure 2.**
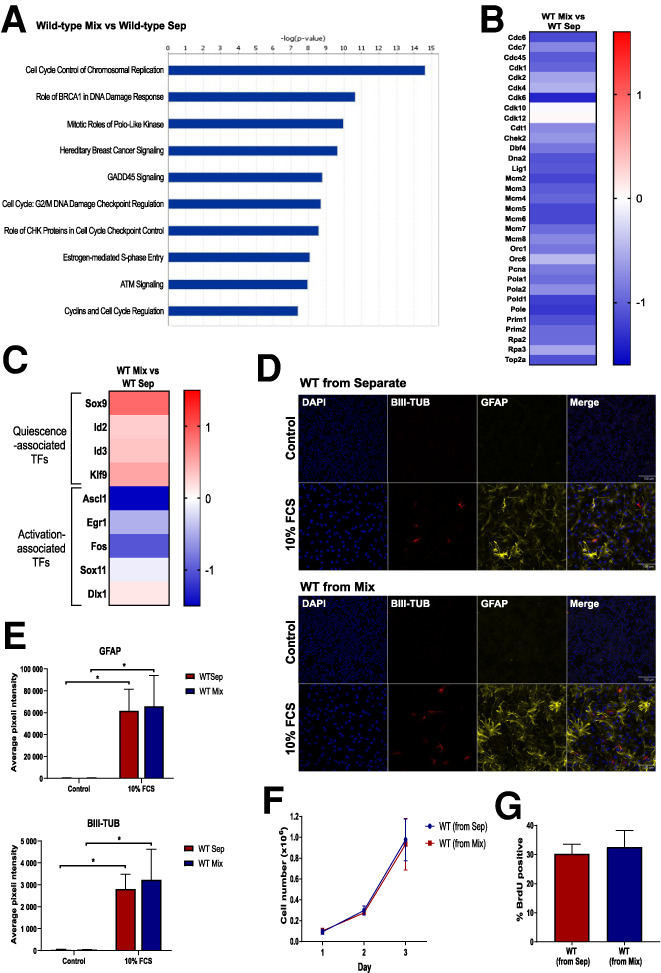
WT-NSCs adopt a quiescent phenotype in the presence of transformed NSCs. (*A–C*) Top canonical pathways (*A*), changes in cell cycle gene expression (*B*), and changes in quiescence and activation-associated gene expression (*C*) in WT-NSCs in coculture versus separate culture revealed by RNA-seq. *N* = 3. (*D*,*E*) Immunofluorescence images (*D*) and quantification (*E*) of WT-NSCs stained with GFAP or BIII-Tubulin after being grown separately or in coculture, then sorted and replated in differentiation-inducing conditions. *N* = 3. Two-way ANOVA followed by Sidak's multiple comparisons test. (*F*,*G*) Growth curves (*F*) and proportion (*G*) of BrdU-positive WT-NSCs after being grown separately or in coculture and then sorted and replated. *N* = 3.

To gain a deeper insight into the mechanism of proliferative arrest of cocultured WT-NSCs, we compared their transcriptional profile with WT-NSCs grown in separate cultures. Cells were isolated by FACS after 5 d of coculture, and RNA-seq was performed. Analysis of differential gene expression with Ingenuity Pathway Analysis, which uses functional annotations and interactions of genes to identify enriched pathways, revealed that the majority of the top canonical pathways enriched in cocultured WT-NSCs were related to cell cycle control and DNA repair (Fig. 2A,B), consistent with the observed proliferation arrest induced by transformed cells.

One possible explanation for this cell cycle arrest is differentiation into postmitotic cell types, such as immature neurons. The expression of NSC markers together with markers for intermediate progenitors and differentiated cell types (Zhang and Jiao 2015) was therefore explored in our RNA-seq data set. No broad down-regulation of classical NSC markers or any up-regulation of differentiation markers was observed in cocultured WT-NSCs compared with their separate counterparts (Supplemental Fig. S2A). Interestingly, we did observe an up-regulation in the expression of the glial markers, *Gfap*, *Glt1*, and *Glast*, which are enriched in quiescent NSCs (Codega et al. 2014; Llorens-Bobadilla et al. 2015). These observations suggest that WT-NSCs are not differentiating, but instead may be driven into a quiescent astrocytic-like state.

To explore this last possibility, we analyzed our RNA-seq data set for the expression of a broader set of quiescent and activated NSC markers. We found that the quiescence-associated transcriptional regulators, *Sox9*, *Id2*, *Id3*, and *Klf9*, which are up-regulated in quiescent SVZ NSCs (Llorens-Bobadilla et al. 2015; Morizur et al. 2018), were all also up-regulated in the cocultured WT-NSCs (Fig. 2C). We also observed that markers of activated NSCs, *Ascl1*, *Egr1*, *Fos*, and *Sox11* (Andersen et al. 2014; Llorens-Bobadilla et al. 2015; Morizur et al. 2018), were down-regulated in these cells. When we compared our RNA-seq data set with a list of transcription factors and cofactors that have been found to be differentially expressed between activated and quiescent SVZ NSCs (Morizur et al. 2018), we found that of the 14 genes enriched in quiescent cells in this study, 10 were up-regulated in cocultured WT-NSCs, and of the 61 genes enriched in activated NSCs, 50 were down-regulated (Supplemental Fig. S2B). Together these findings therefore support the hypothesis that the cocultured NSCs are adopting a quiescent phenotype.

To test whether the quiescent WT-NSCs remain multipotent after coculture with transformed NSCs, we cocultured both these cell types for 5 d, sorted the WT-NSCs, and then replated them in differentiation-inducing conditions. When this was done, we observed that the cocultured WT-NSCs had a similar ability to differentiate into astrocytes, neural progenitors and neurons as separately cultured WT-NSCs did (Fig. 2D,E; Supplemental Fig. S2C,D). This indicates that the cocultured quiescent NSCs retain multipotency.

A key feature of a quiescent phenotype is its reversibility. To test whether the proliferation arrest of WT-NSCs in coculture is reversible, we sorted WT and IE-NSCs after 5 d in coculture and replated them. In parallel, WT-NSCs that had been cultured separately were mixed with transformed cells just before sorting and subsequently replated. Importantly, we observed that previously cocultured WT-NSCs displayed a similar proliferation rate to NSCs that had been separately cultured throughout, as judged by their growth curves and BrdU incorporation (Fig. 2F,G). The reversible nature of the proliferation arrest of cocultured WT-NSCs provides further evidence that they are entering a quiescent state rather than becoming senescent or terminally differentiated.

### Cell contact is required for the inhibitory effect of transformed cells

**Figure 3.**
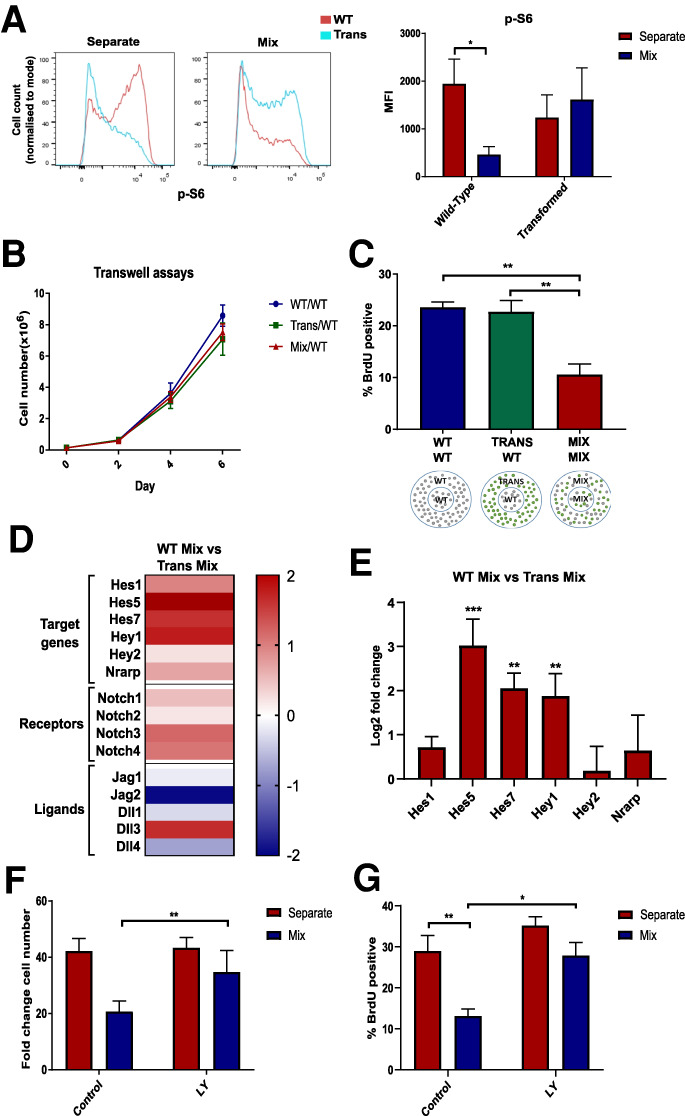
Quiescence of WT-NSCs is induced by direct contact with transformed NSCs and is associated with increased Notch activation. (*A*) Flow cytometry plot showing p-S6 staining in WT and transformed NSCs in separate and coculture and quantification of median fluorescence intensity. *N* = 6, ANOVA followed by Sidak's multiple comparisons test. (*B*) Growth curves of WT-NSCs cultured below transwell inserts with WT, transformed, or mixed cultures. *N* = 3. (*C*) Percentage of BrdU-positive WT-NSCs from the inner ring of the three different cultures shown. *N* = 3. One-way ANOVA followed by Tukey's multiple comparisons test. (*D*,*E*) Heat map showing changes in Notch gene expression after RNA-seq (*D*) and after RT-qPCR in cocultured transformed and WT-NSCs (*E*). *N* = 3. Two-way ANOVA followed by Sidak's multiple comparisons test. (*F*,*G*) Quantification of the fold change in NSC number after separate or coculture with transformed cells (*N* = 8) (*F*) and proportion of BrdU-positive cells with and without addition of LY411575 (*N* = 4) (*G*).

To understand the mechanisms underlying the interaction between WT and transformed NSCs, we first analyzed the possible involvement of the mTOR signaling pathway. mTOR is a metabolic regulator that senses growth factor and nutrient inputs. It is activated in transit amplifying progenitor cells and its inhibition induces quiescence in adult NSCs (Paliouras et al. 2012). Analysis of the expression of ribosomal protein S6 phosphorylation (p-S6), a read-out of mTOR activity indicated that mTOR signaling levels were reduced in cocultured WT-NSCs, not only relative to these same cells in separate culture, but also to IE-NSCs in coculture (Fig. 3A). We next tested whether constitutive mTOR activation was sufficient to prevent these NSCs from entering quiescence in coculture. For this we mutated *Tsc2*, an mTOR repressor in WT-NSCs, using CRISPR-Cas9 targeting (Supplemental Fig. S3A). However, we found that, although this was sufficient to sustain strong mTOR pathway activation, *Tsc2*^−/−^ NSCs still entered a proliferation arrest when cocultured with IE-NSCs (Supplemental Fig. S3B–D). This indicates that mTOR inhibition is not the primary event for the induction of quiescent phenotype of cocultured WT-NSCs.

Next, we investigated the possibility that cell contact is required to induce the growth arrest of WT-NSCs. We first used a transwell assay, where the two cell types are separated by a permeable membrane that allows the exchange of signaling factors in the media while physically separating the cells. When WT-NSCs were separated from IE-NSCs by the transwell, they grew similarly to WT-NSCs in homotypic cultures (Fig. 3B). This suggests that secreted paracrine factors are not sufficient to induce quiescence. We further tested this possibility by using fences, which are metal inserts that divide the well into an inner and outer ring. One cell type is plated in the inner ring and the second in the outer ring (Fig. 3C) and once they are growing adherently, the inserts can be removed, leaving behind a small gap between the two populations. Using this system, we compared the proliferation rate of WT-NSCs that were separated from IE-NSCs with their proliferation when separated from other WT-NSCs or when mixed with IE-NSCs. We found that the rate of BrdU incorporation of the WT-NSCs did not decrease when they were not in physical contact with the IE-NSCs (Fig. 3C). This indicates that cell–cell contact is most likely required for IE-NSCs to inhibit the proliferation of WT-NSCs.

### Transformed cells signal via Notch and Rbpj to induce quiescence in wild-type NCSs

The Notch pathway is a highly conserved signaling pathway requiring cell–cell contact that plays a central role in the regulation of embryonic and adult neurogenesis (Ables et al. 2011; Urbán and Guillemot 2014). To test whether Notch signaling is required to trigger quiescence, we first explored the expression of Notch signaling pathway components in our RNA-seq data. Interestingly, we found that the majority of Notch-related genes were up-regulated in WT-NSCs compared with IE-NSCs, including many Notch targets and receptors, while the Notch ligands appeared to be more highly expressed on IE-NSCs in coculture (Fig. 3D). With the exception of *Jagged1*, this higher expression of Notch ligands in IE-NSCs was not dependent on their coculture with WT-NSCs (Supplemental Fig. S4A), suggesting that cell–cell communication was not driving this initial difference in expression. Analysis by qPCR of sorted samples also revealed that the expression of the Notch target genes *Hes1*, *Hes5*, *Hes7*, *Hey1*, *Hey2*, and *Nrarp* were up-regulated in cocultured WT-NSCs (Fig. 3E). This suggests that Notch pathway activity is higher in cocultured WT-NSCs than in IE-NSCs and raises the possibility that transformed cells may be signaling to WT-NSCs via this pathway.

To test the functional significance of this difference in Notch activation, two complementary γ-secretase inhibitors were used to block Notch signaling. We observed that both LY411575 (LY) and crenigacestat reduced Notch target gene expression in NSCs. Importantly, these also partially rescued the proliferation arrest of cocultured WT-NSCs. The growth curves and BrdU incorporation rates of cocultured NSCs treated with the γ-secretase inhibitors were only slightly lower than their growth is separate culture (Fig. 3F,G; Supplemental Fig. S4B–F). This suggests that Notch signaling is required for the induction of quiescence in NSCs by transformed cells.

**Figure 4.**
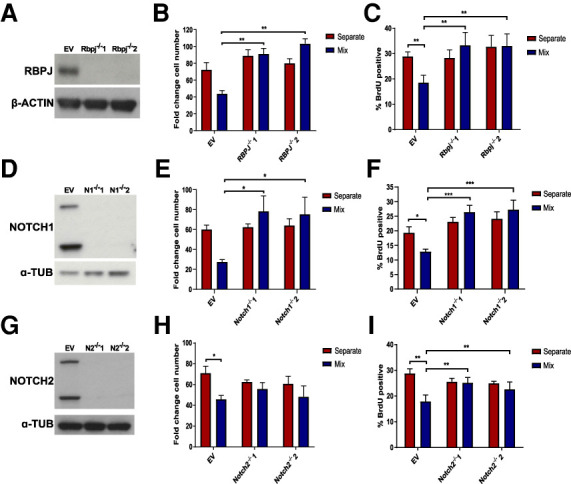
Disruption of Notch signaling rescues WT NSC proliferation in coculture. (*A*,*D*,*G*) Western blot showing absence of RBPJ protein in *Rbpj^−/−^* clones (*A*), NOTCH1 protein in *Notch1^−/−^* clones (*D*), and NOTCH2 protein in *Notch2^−/−^* clones (*G*). (*B*,*C*,*E*,*F*,*H*,*I*) Fold change cell number quantification (*B*,*E*,*H*) and proportion of BrdU-positive cells (*C*,*F*,*I*) in EV and *Rbpj^−/−^* NSCs in separate and coculture with transformed NSCs (*N* = 4, *B*;*N* = 5, *C*), in EV and *Notch1^−/−^* NSCs in separate and coculture with transformed NSCs (*N* = 4, *E*;*N* = 5, *F*), and in EV and *Notch2^−/−^* NSCs in separate and coculture with transformed NSCs (*N* = 4, *H*; *N* = 5, *I*). Two-way ANOVA followed by Sidak's multiple comparisons test.

To further test the requirement for Notch signaling we mutated *Rbpj*, a key effector of this pathway, as well as *Notch1* and *Notch2* by CRISPR/Cas9 targeting (Fig. 4A,D,G). *Rbpj^−/−^, Notch1^−/−^*, or *Notch2^−/−^* NSCs were not susceptible to proliferation arrest when cocultured with IE-NSCs and grew at similar growth rates in separate and coculture (Supplemental Fig. S5A–D). Similarly, the fold change in cell number and BrdU incorporation rates of cocultured *Rbpj^−/−^*, *Notch1^−/−^* and *Notch2^−/−^* NSCs was similar to separate culture and significantly higher than that of WT-NSCs cocultured with IE-NSCs (Fig. 4B,C,E,F,H,I). Interestingly, both the growth curves and final cell counts of *Rbpj^−/−^* NSCs indicated that these cell types even outcompeted IE-NSCs in coculture (Fig. 4B; Supplemental Fig. S5B), suggesting that disrupting Notch signaling may be sufficient to provide NSCs with a competitive advantage against transformed NSCs. The ability of both *Notch1* and *Notch2* deletion to render the NSCs insensitive to proliferation arrest suggests that these receptors both have a role in conveying the quiescence-inducing signal to WT NSCs. This is in contrast to recent studies that have suggested a nonredundant role for Notch2 in promoting NSC quiescence (Engler et al. 2018). It would also suggest that it is not necessary to completely block Notch signaling to prevent growth arrest, reducing the strength of the signal through deletion of a single receptor was sufficient.

To exclude the possibility that Notch signaling is required in transformed NSCs, we mutated *Rbpj* in this cell type (Supplemental Fig. S5E). We observed that IE-NSCs still induced the proliferation arrest of WT-NSCs, as shown by the growth curves, final cell numbers and BrdU incorporation (Supplemental Fig. S5F–H). Therefore, Notch signaling is not required in transformed cells for their growth inhibition of WT-NSCs.

Finally, to test if cells carrying other mutations that induce transformation can also induce quiescence in WT-NSCs we analyzed *Pten*^−/−^; *p53*^−/−^ NSCs, that when mutated in mice induce a glioblastoma-like phenotype (Zheng et al. 2008; Jacques et al. 2010). Mutant NSCs were generated by CRISPR/Cas9 targeting and cultured separately or cocultured with WT-NSCs. Indeed, *Pten*^−/−^; *p53*^−/−^ NSCs were capable of inducing a growth arrest in WT-NSCs (Supplemental Fig. S6). The ability to induce quiescence in neighboring cells is therefore not dependent on the specific IENS model, but may be a common feature of several different types of transformed NSCs.

Altogether, these data demonstrate that transformed cells activate Notch signaling in adjacent wild-type activated NSCs and trigger their transition to quiescence. This observation may have implications for the understanding of gliomagenesis. In humans it has been suggested that adult SVZ cells accumulate low-level driver mutations and give rise to GBM tumors (Lee et al. 2018). This niche has also been suggested to harbor malignant cells away from the tumor mass, which are more resistant to chemotherapy and may therefore act as a reservoir for recurrence (Piccirillo et al. 2015). Furthermore, the SVZ has been shown to be a permissive region for GBM growth, as tumors in contact with this region are associated with decreased patient survival and increased recurrence (Khalifa et al. 2017; Mistry et al. 2017).

Our studies using SVZ-derived wild-type and transformed NSCs demonstrate that transformed cells provide a negative feedback via Notch signaling to surrounding normal NSCs, which cause them to exit their activated state and re-enter quiescence. By this mechanism, oncogenic NSCs outcompete normal NSCs and ensure their preferential self-renewal and differentiation, therefore increasing both their cell number and their likelihood of giving rise to progenitor cells and exiting the niche. This competitive advantage may be an important early step in the development of glioma and GBM. Confirming and tracking quiescent NSCs in vivo and establishing how this competition mechanism operates in the complex milieu of the SVZ niche are important future directions to understand the aetiology of GBM.

## Materials and methods

### Neural stem cell cultures

Adult murine NSCs were derived from the SVZ by the protocol described in Conti et al. (2005) and Pollard et al. (2006). Transformed *Ink4a/Arf^−/−^*; EGFRvIII NSCs (IE-NSCs) were previously generated by the Maarten van Lohuizen laboratory (Netherlands Cancer Institute [NKI]). NSCs were isolated from *Ink4a/Arf^−/−^* mice and transduced with EGFRvIII pMSCV retrovirus (Bruggeman et al. 2007).

### Coculture assays

Forty-thousand cells per well were seeded in a 24-well plate either separately or as a 50:50 mix of WT and IE-NSCs. The proportion of each cell type was assessed by determining the percentage of GFP-positive cells by flow cytometry performed on an LSR-II Analyzer (BD Biosciences).

### Differentiation of NSCs

Sorting was performed by FACS at day 5 of the 6-d assay for three biological repeats. Cells were seeded onto coverslips in NSC medium for 24 h following sorting. Medium was then replaced for either NSC media with 10% FCS or NSC media without EGF addition. Medium was replaced every other day and cells were fixed after 4 d in FCS or 6 d without EGF.

### Flow cytometry immunolabeling

Cells were fixed in 2% formaldehyde and permeabilized in ice-cold 90% methanol. For BrdU analysis, cells were incubated with BrdU for 2 h prior to fixation. Cells were then incubated with DNaseI prior to blocking. Primary antibodies (p-S6, cleaved Caspase3, or BrdU; 1:200; CST) were detected with Alexa Fluor 546 secondary antibodies (Molecular Probes). Flow cytometry was performed on an LSR-II Analyzer (BD Biosciences) and data were analyzed with the FlowJo software.

### RNA sequencing analysis

Sorting was performed by FACS at day 5 of the 6-d assay for three biological repeats. mRNA was isolated, fragmented, converted to cDNA, and ligated to Illumina adapters. Sequencing was performed using the HiSeq 4000 system (Illumina). Differential expression was determined using the DESeq2 package (Love et al. 2014) and the resulting gene sets were analyzed using IPA software (Qiagen) (Krämer et al. 2014). A “core analysis” was performed for each comparison on genes differentially expressed with a false discovery rate of <10%.

### CRISPR gene targeting of NSCs

gRNAs for targeting of *Rbpj, Notch1*, *Notch2*, and *P53* were cloned into the pX330-U6-Chimeric_BB-CBh-hSpCas9 (PX330) expression plasmid (gift from Feng Zhang, Addgene plasmid 42230) as previously described (Ran et al. 2013). Details of *Tsc2^−/−^* NSC generation are in the Supplemental Material and gRNA sequences in Supplemental Table S1. NSCs were transfected with 2 µg of plasmid DNA and 0.125 µg of Puro-pPYCAGIP vector or linear hygromycin marker (Clontech 631625) by nucleofection (Lonza AMAXA 2B). Selection was performed from 48 h posttransfection until the emergence of resistant colonies. *Pten^−/−^* NSCs were generated as described in Bressan et al. (2017) and then transfected with gRNAs targeting *P53* as described above.

### Quantification and statistical analysis

Statistical analysis and data representation were performed using GraphPad Prism software. Statistical methods used and sample size (*n*) are indicated in the relevant figure legends. Adjusted *P* values are displayed as *P* < 0.05 (*), *P* < 0.01 (**), and *P* < 0.001 (***).

### Data availability

The raw data for the RNA sequencing of wild-type and transformed NSCs have been deposited in the ArrayExpress database under accession number E-MTAB-8580.

### Competing interest statement

Steven Pollard is founder and shareholder of Cellinta.

## Supplementary Material

Supplemental Material
