# Supplementary material for: Glioblastoma stem cells induce quiescence in surrounding neural stem cells via Notch signaling
Source: Genes Dev. 2020 Dec 1;34(23-24):1599–604. doi: 10.1101/gad.336917.120 (PMC7706704; doi:10.1101/gad.336917.120)
Supplement: Supplemental Material [file supp_34_23-24_1599__index.html]

Glioblastoma stem cells induce quiescence in surrounding neural stem cells via Notch signaling — Supplemental Material 

# Glioblastoma stem cells induce quiescence in surrounding neural stem cells via Notch signaling

## Supplemental Material

- Supplemental\_Fig\_S1.pdf
- Supplemental\_Fig\_S2.pdf
- Supplemental\_Fig\_S3.pdf
- Supplemental\_Fig\_S4.pdf
- Supplemental\_Fig\_S5.pdf
- Supplemental\_Fig\_S6.pdf
- Supplemental\_Figure\_Legends.docx
- Supplemental\_Tables.docx
- Supplemental\_Methods.docx
