# Supplementary figures and images for: Glioblastoma stem cells induce quiescence in surrounding neural stem cells via Notch signaling
Source: Genes Dev. 2020 Dec 1;34(23-24):1599–604. doi: 10.1101/gad.336917.120 (PMC7706704; doi:10.1101/gad.336917.120)

# Supplementary Figure 1

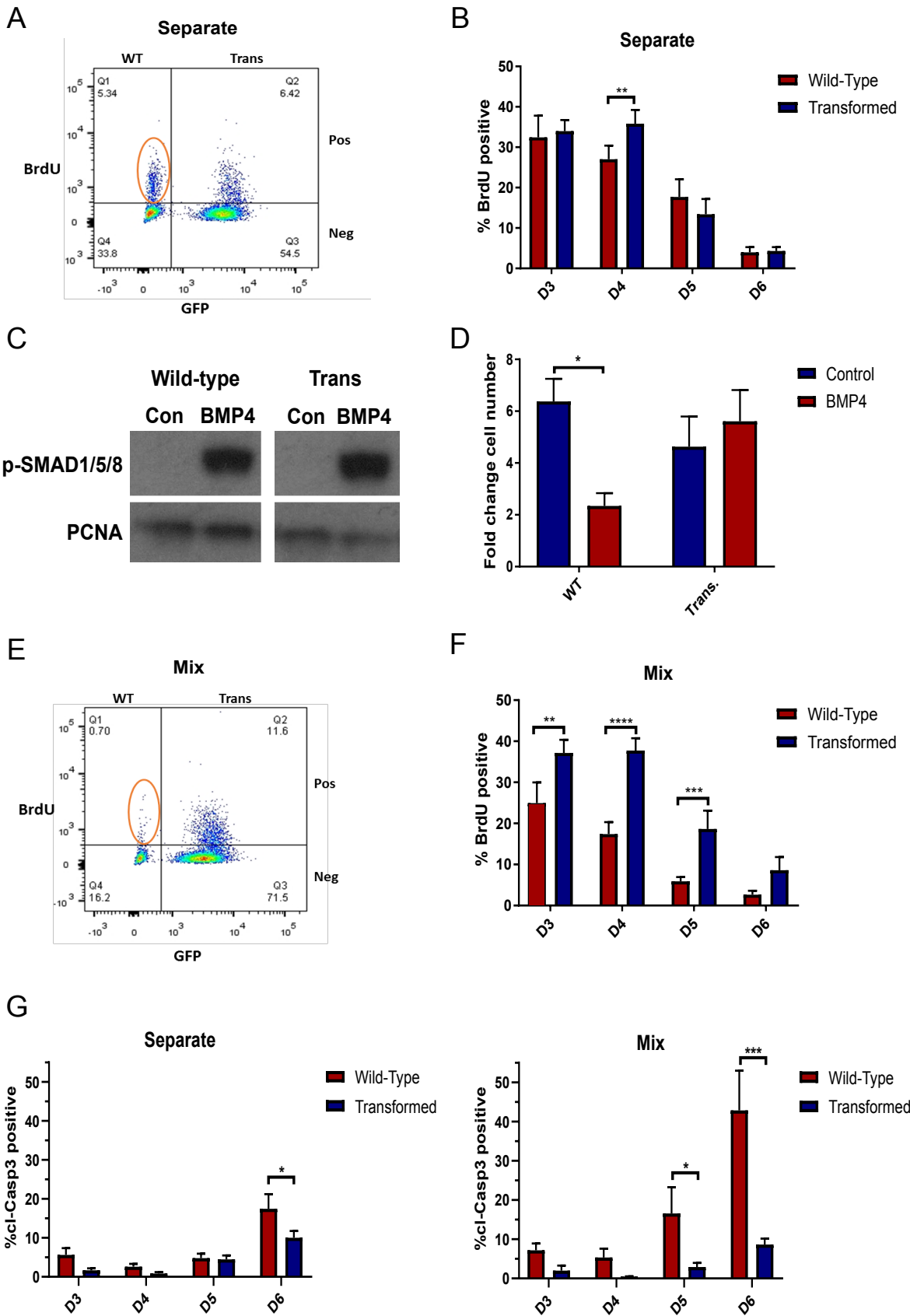

Supplement: Supplemental Material [file supp_gad.336917.120_Supplemental_Fig_S1.pdf]

**A**

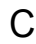

Control

EGF

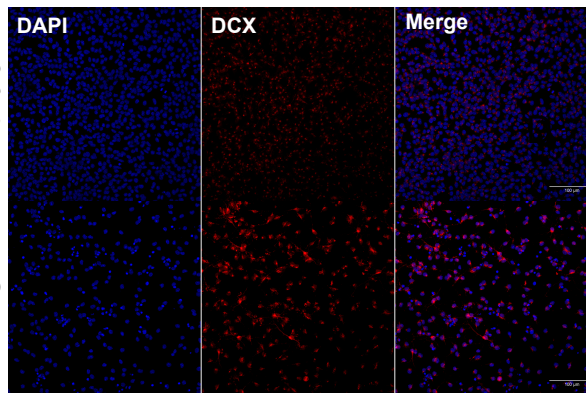

Control

EGF

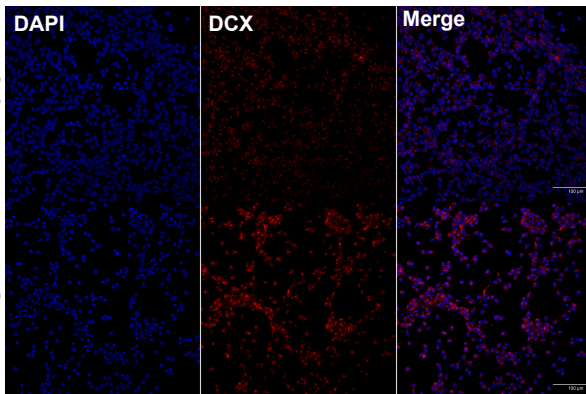

B

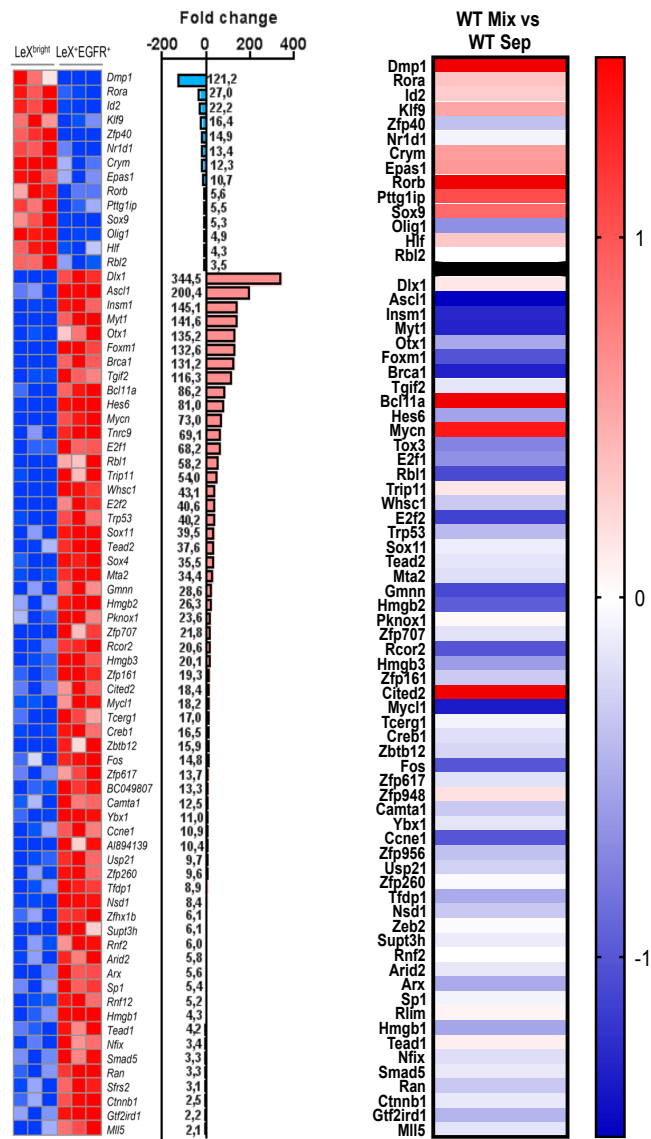

E

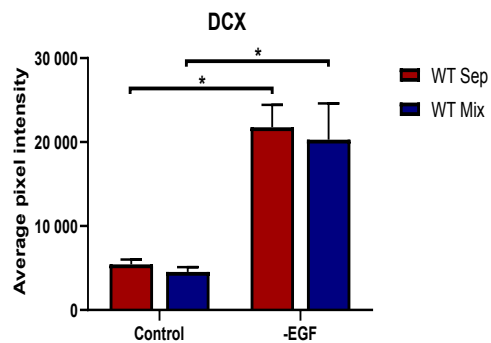

Supplement: Supplemental Material [file supp_gad.336917.120_Supplemental_Figure_2_.pdf]

# Supplementary Figure 3

A

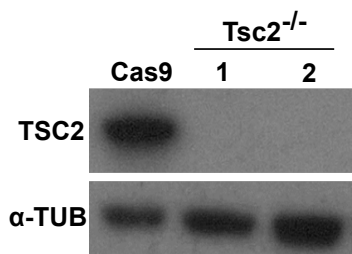

B

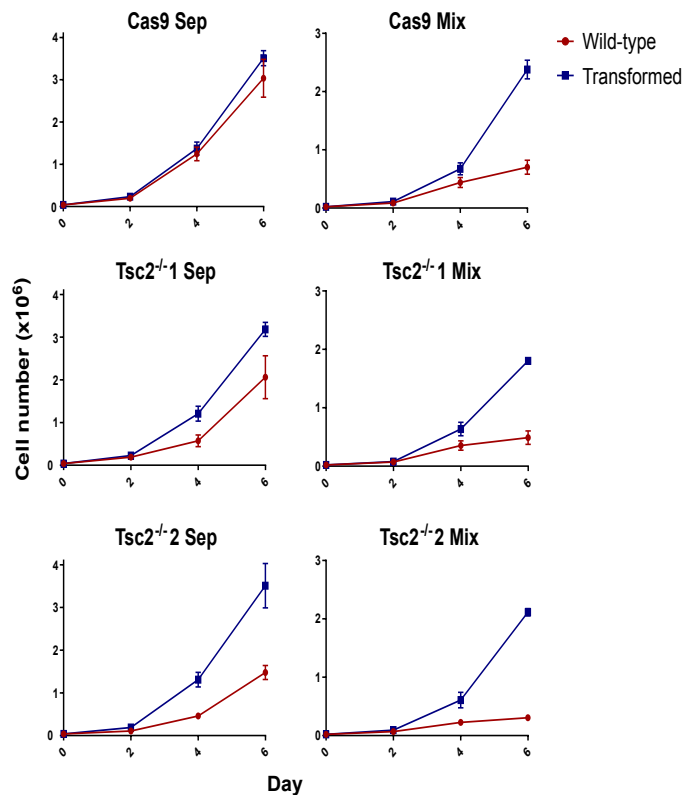

C

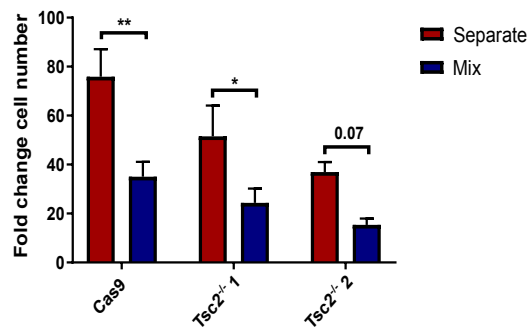

D

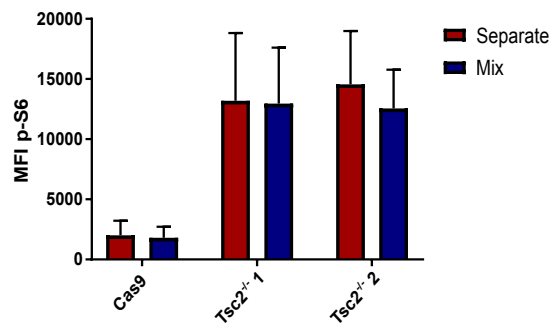

Supplement: Supplemental Material [file supp_gad.336917.120_Supplemental_Fig_S3.pdf]

# Supplementary Figure 4

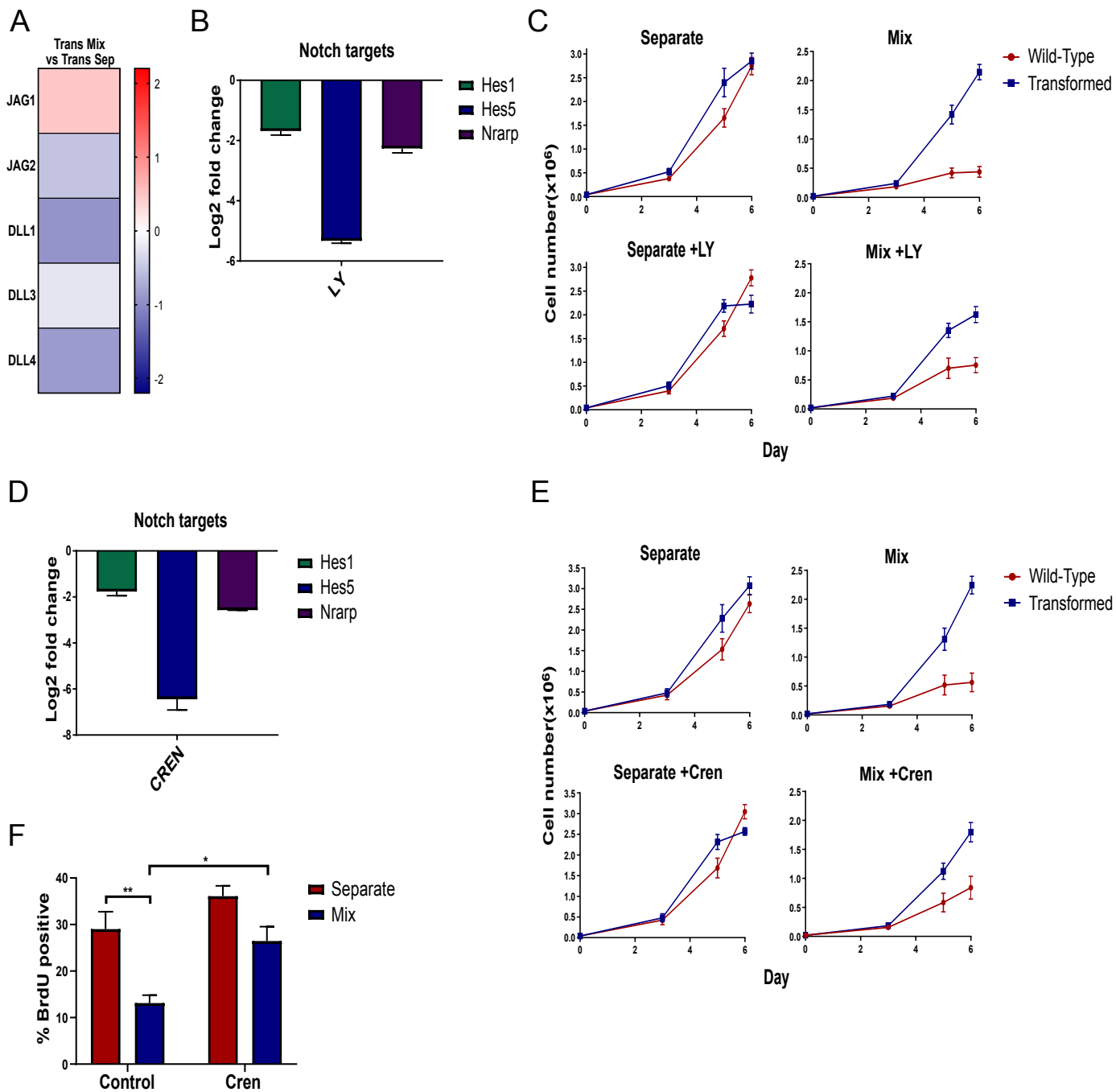

Supplement: Supplemental Material [file supp_gad.336917.120_Supplemental_Fig_S4.pdf]

# Supplementary Figure 5

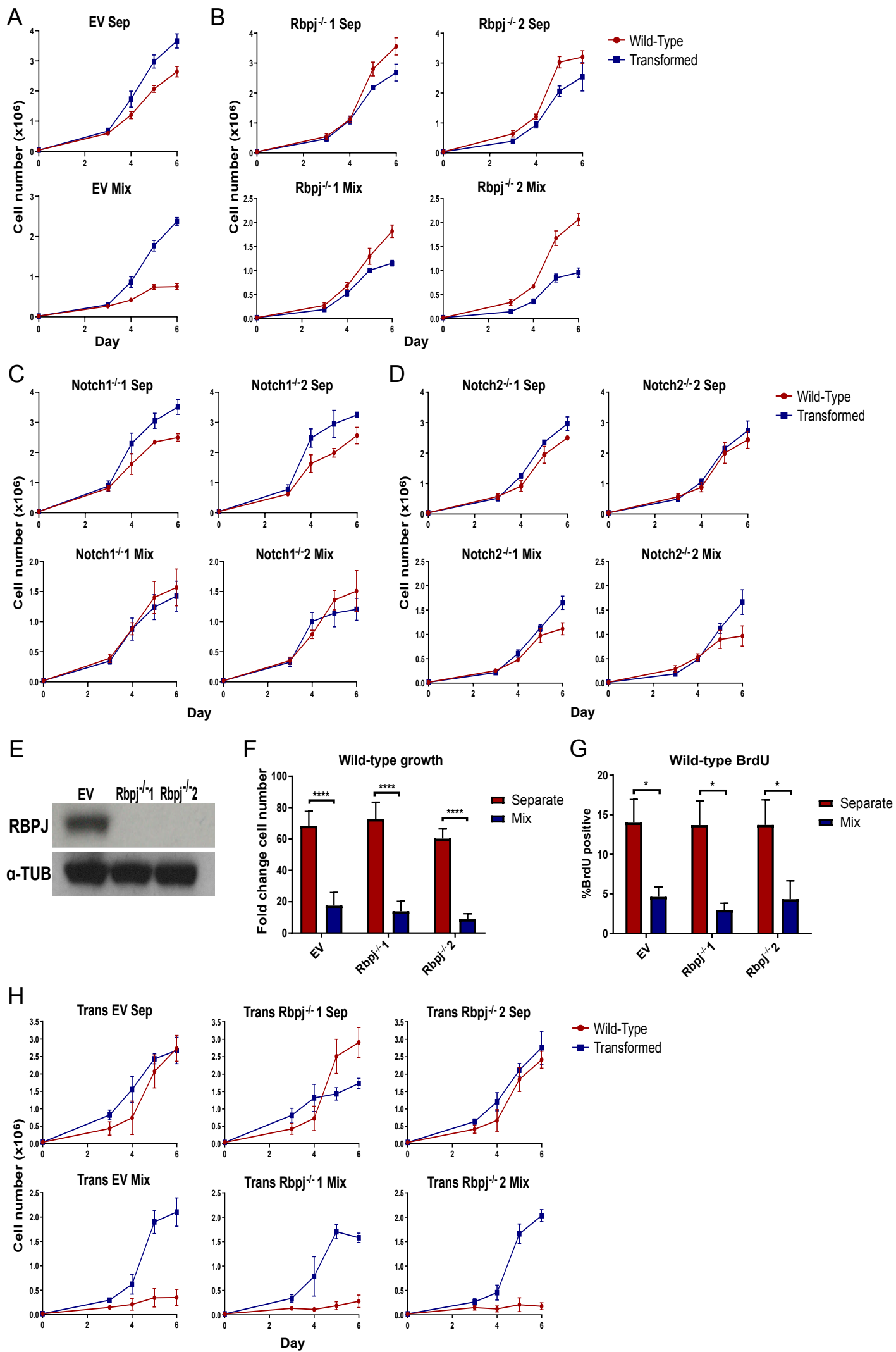

Supplement: Supplemental Material [file supp_gad.336917.120_Supplemental_Fig_S5.pdf]

# Supplementary Figure 6

A

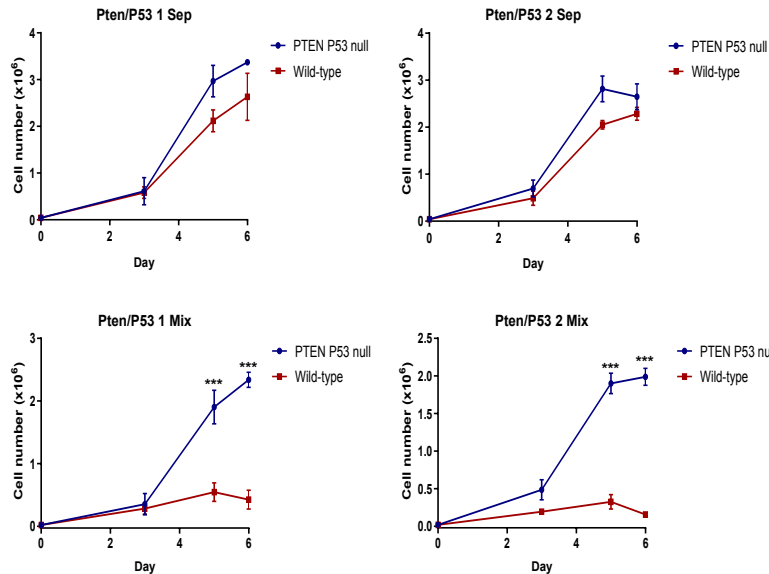

B

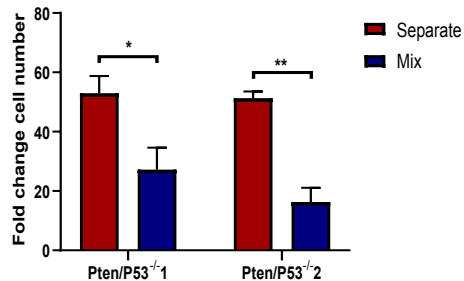

C

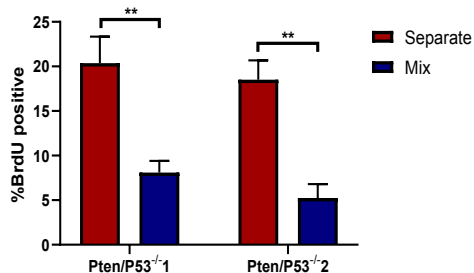

Supplement: Supplemental Material [file supp_gad.336917.120_Supplemental_Fig_S6.pdf]
