# Supplementary material for: Glioblastoma stem cells induce quiescence in surrounding neural stem cells via Notch signaling
Source: Genes Dev. 2020 Dec 1;34(23-24):1599–604. doi: 10.1101/gad.336917.120 (PMC7706704; doi:10.1101/gad.336917.120)
Supplement: Supplemental Material [file supp_gad.336917.120_Supplemental_Figure_Legends.docx]

**Supplementary Figure Legends**

**Supplemental Figure 1**

**(A)** Representative flow cytometry plot of BrdU staining in separately cultured NSCs at day 5. **(B)** Time-course of BrdU staining throughout the assay in separate culture. N=5, ANOVA followed by Sidak’s multiple comparisons test. **(C)** Fold change cell number quantification of NSCs cultured in the presence or absence of 50ng/ml BMP4. N=4, 2-way ANOVA followed by Sidak’s multiple comparisons test. **(D)** Western blot showing increased p-SMAD1/5/8 expression in NSCs treated with 50ng/ml BMP4 for 24 hours relative to control NSCs (Con). **(E)** Representative flow cytometry plot of BrdU staining in co-cultured NSCs at day 5. **(F)** Time-course of BrdU staining throughout the assay in co-culture showing reduction in BrdU incorporation in WT-NSCs. N=5, ANOVA followed by Sidak’s multiple comparisons test. (**G)** Time-course of cleaved-caspase-3 expression showing increase in co-cultured WT NSCs. N=3, ANOVA followed by Sidak’s multiple comparisons test.

**Supplemental Figure 2**

**(A)** Changes in gene expression of classical marker genes in the ‘WT Mix vs WT Sep’ comparison. GFAP log_2_ fold change is off the scale at 5.4. **(B)** Comparison to published transcriptional analysis of quiescent and activated NSCs. Left - Transcription factors and co-factors differentially expressed in quiescent and activated NSCs from (Morizur et al., 2018). Right – Heat map showing the expression of the genes identified by Morizur et al in the comparison of WT NSCs in separate (WT Sep) or co-culture (WT Mix). Genes enriched in quiescent NSCs were broadly upregulated in co-cultured WT NSCs, while those enriched in activated NSCs were broadly downregulated. **(C)** Immunofluorescence images and **(D)** quantification of WT-NSCs stained with Doublecortin after being grown separately or in co-culture, then sorted and re-plated in differentiation inducing conditions. N=3, 2-way ANOVA followed by Sidak’s multiple comparisons test.

**Supplemental Figure 3**

**(A)** Western blot showing absence of TSC2 expression in *Tsc2^-/-^* clones. **(B)** Growth curves of transformed NSCs and *Tsc2^-/-^* or Cas9 control NSCs in separate or co-culture. *Tsc2^-/-^* clones grow more slowly than control and still show reduced growth in co-culture. N=4. **(C)** Fold change cell number quantification of growth curves. N=4, 2-way ANOVA followed by Sidak’s multiple comparisons test. **(D)** Expression of p-S6 in Cas9 and *Tsc2^-/-^* clones as determined by flow cytometry. mTOR activation is increased in *Tsc2^-/-^* NSCs. N=3.

**Supplemental Figure 4**

**(A)** Heat map of RNA-seq analysis showing changes in Notch ligand gene expression in IE-NSCs. **(B)** RT-qPCR analysis of the Notch target genes, *Hes1*, *Hes5* and *Nrarp* in WT NSCs treated with 2µM LY411575. Log_2_ fold change relative to control NSCs. N=3. **(C)** Growth curves of WT and transformed NSCs cultured in the standard assay with or without LY411575 addition from day 3. **(D)** RT-qPCR analysis of the Notch target genes, *Hes1*, *Hes5* and *Nrarp* in WT NSCs treated with 1µM crenigacestat. Log_2_ fold change relative to control NSCs. N=3. **(E)** Growth curves of WT and transformed NSCs cultured with or without crenigacestat addition from day 3. N=8, 2-way ANOVA followed by Sidak’s multiple comparisons test. **(F)** Proportion of BrdU positive cells following a 24-hour incubation with crenigacestat. N=4, 2-way ANOVA with Sidak’s multiple comparisons test. The γ-secretase inhibitor crenigacestat also increases WT NSC proliferation in co-culture.

**Supplemental Figure 5**

**(A, B, C and D)** Growth curves of control EV NSCs (A), *Rbpj^-/-^* NSCs (B), *Notch1^-/-^* NSCs (C), and *Notch2^-/-^* NSCs (D) cultured separately or in co-culture with transformed NSCs. N>4. **(E)** Western blot showing absence of RBPJ protein from *Rbpj^-/-^* transformed NSCs. **(F)** Fold change cell number of WT NSCs in separate or co-culture with EV or *Rbpj^-/-^* transformed NSCs. N=5, 2-way ANOVA followed by Sidak’s multiple comparisons test. **(G)** Proportion of BrdU-positive WT NSCs in separate or co-culture with EV or *Rbpj^-/-^* transformed cells. N=3, 2-way ANOVA followed by Sidak’s multiple comparisons test. **(H)** Growth curves of transformed EV and *Rbpj^-/-^* clones cultured separately or in co-culture with WT NSCs. Both *Rbpj^-/-^* clones suppress the proliferation of WT NSCs to a comparable degree to the EV control transformed cells.

**Supplemental Figure 6**

**(A)** Growth curves of control EV NSCs and *Pten^-/-^; P53^-/-^* NSCs cultured separately or co-cultured. N=3, ANOVA followed by Sidak’s multiple comparisons test. **(B)** Fold change cell number of WT NSCs in separate or co-culture with *Pten^-/-^; P53^-/-^* NSCs. N=3, ANOVA followed by Sidak’s multiple comparisons test. **(C)** Proportion of BrdU-positive WT NSCs in separate or co-culture with *Pten^-/-^; P53^-/-^* NSCs. N=3, two-way ANOVA followed by Sidak’s multiple comparisons test.
