# Supplementary material for: Glioblastoma stem cells induce quiescence in surrounding neural stem cells via Notch signaling
Source: Genes Dev. 2020 Dec 1;34(23-24):1599–604. doi: 10.1101/gad.336917.120 (PMC7706704; doi:10.1101/gad.336917.120)
Supplement: Supplemental Material [file supp_gad.336917.120_Supplemental_Tables_.docx]

**Supplemental Tables**

**Supplemental Table 1. gRNA sequences for deletion**

| **Target gene** | **gRNA1** | **gRNA2** |
| --- | --- | --- |
| Rbpj | TGCAGTGGACGACGACGAGT | GTGGACGACGACGAGTCGGA |
| Notch1 | GGCGTTCAGTGCCTACACAA |  |
| Notch2 | GTCCACCTGCATTGACCGCG | AAGTGCATCGATCACCCGAA |
| Tsc2 | GAAGGCCGGCCTACCTCATT | GCATGGCTCTTACAGGTACA |

**Supplemental Table 2. Primers used in qPCR**

| **Target gene** | **Forward primer** | **Reverse primer** |
| --- | --- | --- |
| Hes1 | GGAAATGACTGTGAAGCACCTCC | GAAGCGGGTCACCTCGTTCATG |
| Hes5 | GCCCGGGGTTCTATGATATT | GAGTTCGGCCTTCACAAAAG |
| Hes7 | CATCAACCGCAGCCTAGAAGAG | CACGGCGAACTCCAGTATCTCC |
| Hey1 | CCAACGACATCGTCCCAGGTTT | CTGCTTCTCAAAGGCACTGGGT |
| Hey2 | TGAAGATGCTCCAGGCTACAGG | CCTTCCACTGAGCTTAGGTACC |
| Nrarp | CAGACAGCACTACACCAGTCAG | CCGAAAGCGGCGATGTGTAGC |
| β-actin | GGCACCACACCTTCTACAATG | GGGGTGTTGAAGGTCTCAAAC |
