# Supplementary material for: Glioblastoma stem cells induce quiescence in surrounding neural stem cells via Notch signaling
Source: Genes Dev. 2020 Dec 1;34(23-24):1599–604. doi: 10.1101/gad.336917.120 (PMC7706704; doi:10.1101/gad.336917.120)
Supplement: Supplemental Material [file supp_gad.336917.120_Supplemental_Methods_.docx]

**Supplemental Methods**

**Transwell and Fence assays**

For transwell assays, 140,000 NSCs were seeded on and below PLL-coated cell culture inserts (Merck Millipore). The growth of WT NSCs seeded below WT or IE-NSCs or a 50:50 mix was evaluated over 6 days.

For Fence assays fences (Aix-Scientifics) were placed in each well of a PLL-coated 24-well plate. 100,000 NSCs were seeded in the inner ring and 400,000 in the outer ring. The fences were removed the following day and 10µM BrdU was added to the wells 48 hours after seeding for 2 hours. The fences were then replaced into the well and the cells in the inner ring were harvested and fixed in 2% formaldehyde. BrdU expression was analysed by flow cytometry as described below.

**Flow cytometry immunolabelling (extended details)**

Cells were fixed in 2% formaldehyde and permeabilised in ice-cold 90% methanol. Cells were incubated with the primary antibody (p-S6, cleaved-Caspase3 or BrdU, CST 1:200) for 1 hour. AlexaFluor 546 secondary antibodies (Molecular Probes) were used for detection. Controls with the primary antibody omitted were used to gate positive and negative cells. Flow cytometry was performed on an LSR-II Analyser (BD Biosciences) with FACS Diva software (BD Biosciences) and data were analysed using FlowJo software. For BrdU analysis, NSCs were incubated for two hours with 10µM BrdU prior to fixation. Before blocking, NSCs were then incubated for 45 minutes at 37°C in DNase I solution consisting of 20µl DNase I (Promega RQ1) in 250µl DNase buffer solution. The immunolabelling protocol was then followed as detailed above.

**Western blot analysis**

Cells were lysed directly in Laemmli buffer (50mM Tris-HCl (pH 6.8), 2% SDS, 10% glycerol in ddH_2_O) and heated at 95°C for 10 minutes. Protein quantification was carried out using a BCA Protein Assay kit (Pierce) according to the manufacturer’s instructions. 15-20μg denatured protein from each sample was run in XT sample buffer (BioRad) with 0.25% β-mercaptoethanol on 10% SDS-PAGE Bis-Tris gels (pre-cast BioRad). Proteins were then blotted onto PVDF membranes (Thermo Fisher Scientific). Primary antibody incubation was overnight at 4^o^C in TBST with 5% BSA. All primary antibodies were from CST and were diluted 1:1000. Horseradish peroxidase-conjugated secondary antibodies (Abcam) diluted 1:5000 were used for detection by the addition of an enhanced chemiluminescence (ECL) substrate (Promega). Western blot quantification was performed using Fiji software (Schindelin et al. 2012).

**Immunocytochemistry**

Cells were fixed in 4% PFA for 10 minutes and blocked for one hour in 5% goat serum and 0.3% Triton. Primary antibodies (GFAP 1:300 Sigma, Beta-III-Tubulin 1:200 CST, DCX 1:200 Abcam) were incubated overnight at 4°C and secondary antibodies (AlexaFluor 1:600 ThermoFisher) were incubated with the coverslips for one hour. Coverslips were mounted with Vectashield (Vector Labs) and imaged on a ZEISS LSM-780 inverted confocal microscope. Image processing and quantification of pixel intensity was performed on FIJI.

**RNA Extraction and Quantitative RT-PCR**

RNA was extracted with the RNeasy mini kit (Qiagen) and SuperScript III reverse transcriptase (Thermo Fisher Scientific) was used for cDNA synthesis according to manufacturer’s instructions. RT-qPCR was performed using SYBR-Green Mastermix with ROX reference dye (Sigma). A list of all primer sequences can be found in Supplementary Table S2. RT-qPCR was performed on an ABI 7900HT Fast Real Time PCR machine (Applied Biosystems) and analysed on SDS Biosystem software. Relative gene expression was calculated using the ΔΔCt method normalised to β-actin expression (Livak and Schmittgen 2001).

**RNA-sequencing analysis (extended details)**

Sorting was performed by FACS at day 5 of the 6-day assay for 3 biological repeats. Cells were lysed and RNA extracted with the RNeasy mini kit (Qiagen). RNA samples were quantified using a Qubit fluorometer (Thermo Fisher Scientific) and the quality assessed by TapeStation electrophoresis (Agilent). mRNA was isolated using oligo dT beads. mRNA was then fragmented, converted to cDNA and ligated to Illumina adapters. Following sample indexing, the quality of cDNA libraries was also assessed by TapeStation. Sequencing was performed using the HiSeq 4000 system (Illumina). Sequencing reads were aligned using TopHat2 (Kim et al. 2013) and differential expression was analysed using the DESeq2 package (Love et al. 2014). The resulting gene sets were analysed using Ingenuity Pathway Analysis (IPA) software (Qiagen)(Kramer et al. 2014). A ‘Core Analysis’ was performed for each comparison on genes differentially expressed with a false discovery rate (FDR) of less than 10%.

**CRISPR gene targeting of NSCs (extended details)**

gRNAs for targeting of *Rbpj, Notch1*, *Notch2* and *P53^-/-^* were cloned into the pX330-U6-Chimeric_BB-CBh-hSpCas9 (PX330) expression plasmid (gift from Feng Zhang, Addgene plasmid #42230) as previously described (Ran et al. 2013). gRNAs for targeting *Tsc2* were cloned into gRNA expression plasmids (gift from George Church, Addgene plasmid #41824) (Mali et al. 2013). These were then co-transfected with an hCas9 plasmid (gift from George Church, Addgene plasmid #41815). gRNA sequences can be found in Supplemental Table S1. NSCs were transfected with plasmids by nucleofection (AMAXA 2B, Lonza) using programme A-033. 2-3x10^6^ cells were resuspended in 100µl mouse neural stem cell nucleofector buffer (Lonza) with 2µg plasmid DNA. NSCs were either transfected with 2ug PX330 vector containing gRNAs targeted to *Rbpj*, *Notch1*, *Notch2* and *P53* or 0.6µg each of hCas9 and the two gRNA expression plasmids containing the *Tsc2*-targeting gRNAs. NSCs were also co-transfected with 0.125ug of Puro-pPyCAGIP vector or linear hygromycin marker (Clontech 631625) for selection. Empty vector or Cas9 only controls were also performed. Cells were recovered post-transfection with pre-warmed NSC growth media into a 10cm^2^ dish. Selection with 0.25ug/ml puromycin or 250ug/ml hygromycin was performed from 48 hours post-transfection until the emergence of resistant colonies. These were then picked manually with a p20 pipette and transferred to a 48 well plate for expansion and analysis. *Pten^-/-^* NSCs were generated as described in (Bressan et al. 2017) and then transfected with gRNAs targeting *P53* as described above.

**Quantification and Statistical Analysis**

Statistical analysis and data representation were performed using GraphPad Prism software. Statistical methods used are indicated in the relevant figure legends. The sample size (n) is described in the figure legends and refers to the number of independent replicate experiments performed. Adjusted p values are displayed as * p<0.05, ** p<0.01 and *** p<0.001.

**Data availability**

The raw data for the RNA-sequencing of wild-type and transformed NSCs have been deposited in the arrayexpress database under accession number E-MTAB-8580.

**References**

Bressan RB, Dewari PS, Kalantzaki M, Gangoso E, Matjusaitis M, Garcia-Diaz C, Blin C, Grant V, Bulstrode H, Gogolok S et al. 2017. Efficient CRISPR/Cas9-assisted gene targeting enables rapid and precise genetic manipulation of mammalian neural stem cells. *Development* **144**: 635-648.

Kim D, Pertea G, Trapnell C, Pimentel H, Kelley R, Salzberg SL. 2013. TopHat2: accurate alignment of transcriptomes in the presence of insertions, deletions and gene fusions. *Genome Biol* **14**: R36.

Kramer A, Green J, Pollard J, Jr., Tugendreich S. 2014. Causal analysis approaches in Ingenuity Pathway Analysis. *Bioinformatics* **30**: 523-530.

Livak KJ, Schmittgen TD. 2001. Analysis of relative gene expression data using real-time quantitative PCR and the 2(-Delta Delta C(T)) Method. *Methods* **25**: 402-408.

Love MI, Huber W, Anders S. 2014. Moderated estimation of fold change and dispersion for RNA-seq data with DESeq2. *Genome Biol* **15**: 550.

Mali P, Yang L, Esvelt KM, Aach J, Guell M, DiCarlo JE, Norville JE, Church GM. 2013. RNA-guided human genome engineering via Cas9. *Science* **339**: 823-826.

Ran FA, Hsu PD, Wright J, Agarwala V, Scott DA, Zhang F. 2013. Genome engineering using the CRISPR-Cas9 system. *Nat Protoc* **8**: 2281-2308.

Schindelin J, Arganda-Carreras I, Frise E, Kaynig V, Longair M, Pietzsch T, Preibisch S, Rueden C, Saalfeld S, Schmid B et al. 2012. Fiji: an open-source platform for biological-image analysis. *Nat Methods* **9**: 676-682.
